# The clinical potential of articular cartilage-derived progenitor cells: a systematic review

**DOI:** 10.1038/s41536-021-00203-6

**Published:** 2022-01-10

**Authors:** Margot Rikkers, Jasmijn V. Korpershoek, Riccardo Levato, Jos Malda, Lucienne A. Vonk

**Affiliations:** 1 Department of Orthopaedics, University Medical Center Utrecht, Utrecht University, Utrecht, The Netherlands; 2 Department of Clinical Sciences, Faculty of Veterinary Medicine, Utrecht University, Utrecht, The Netherlands; 3 Present Address: CO.DON AG, Teltow, Germany

**Keywords:** Translational research, Regeneration, Adult stem cells, Stem-cell research

## Abstract

Over the past two decades, evidence has emerged for the existence of a distinct population of endogenous progenitor cells in adult articular cartilage, predominantly referred to as articular cartilage-derived progenitor cells (ACPCs). This progenitor population can be isolated from articular cartilage of a broad range of species, including human, equine, and bovine cartilage. In vitro, ACPCs possess mesenchymal stromal cell (MSC)-like characteristics, such as colony forming potential, extensive proliferation, and multilineage potential. Contrary to bone marrow-derived MSCs, ACPCs exhibit no signs of hypertrophic differentiation and therefore hold potential for cartilage repair. As no unique cell marker or marker set has been established to specifically identify ACPCs, isolation and characterization protocols vary greatly. This systematic review summarizes the state-of-the-art research on this promising cell type for use in cartilage repair therapies. It provides an overview of the available literature on endogenous progenitor cells in adult articular cartilage and specifically compares identification of these cell populations in healthy and osteoarthritic (OA) cartilage, isolation procedures, in vitro characterization, and advantages over other cell types used for cartilage repair. The methods for the systematic review were prospectively registered in PROSPERO (CRD42020184775).

## Introduction

Hyaline cartilage facilitates smooth movement of articular joints and transmission of mechanical forces. The mechanical strength of cartilage tissue is provided by the combination of highly organized collagen arcades and negatively charged proteoglycans that draw water into the tissue^1^. Persisting damage to this structural organization leads to a change in the distribution of forces and loss in mechanical strength^2^. Cartilage injury can be post-traumatic, where defects are generally isolated, or it can occur during the progression of osteoarthritis (OA) where defects can emerge simultaneously. Both focal defects and OA impair quality of life leading to pain, reduced mobility, and disability^3,4^. As the healthy articular cartilage is an avascular tissue, its endogenous healing capacity is limited.

Adult chondrocytes, the cells residing in articular cartilage, are used to treat cartilage defects in autologous chondrocyte implantation^5^. Due to the low cell density in cartilage, chondrocytes are culture-expanded to obtain a sufficient number of cells for treatment. Expansion of chondrocytes is limited in population doublings^6^, as they tend to acquire a fibroblastic appearance and lose their chondrogenic phenotype^7,8^, before becoming senescent. Alternatively, the use of mesenchymal stromal cells (MSCs) for cartilage repair has been evaluated extensively in clinical studies^9^. Despite their capacity to generate cartilaginous tissue, MSCs have a tendency for differentiation into hypertrophic chondrocytes and subsequent endochondral ossification^10^. In contrast, MSCs are suggested to have chondro-inductive effects when combined with autologous chondrons for the treatment of focal cartilage defects^11^.

A distinct population of endogenous progenitor cells that resides in articular cartilage, named articular cartilage-derived progenitor cells (ACPCs), has been described in the last two decades^12–15^. The key in vitro characteristics of ACPCs include stem cell-like properties such as clonal expansion, extensive proliferation, and differentiation potential into multiple mesenchymal lineages, including the chondrogenic lineage. ACPCs were first identified in bovine cartilage^16^, and later also in different species, including equine^7,13^ and human cartilage^17,18^. Interestingly, ACPCs were shown not to upregulate type X collagen gene expression in vitro, a marker for hypertrophic differentiation during redifferentiation, contrary to MSCs^7,13^. The use of an endogenous cartilage progenitor cell population for treatment of cartilage defects and tissue engineering purposes therefore seems favorable over the use of other cell types^14,19,20^. Yet, isolation protocols and specific characterization for these cells differ greatly amongst researchers. In addition, a wide range of terms is being used to name the cells, like chondrogenic progenitor cells, cartilage stem cells, mesenchymal progenitor cells, or cartilage-derived stem/progenitor cells. For clarity, this review refers to ACPCs to address all endogenous progenitor populations identified in adult hyaline cartilage and characterized in vitro.

The purpose of this review is to systematically evaluate the available literature on ACPCs derived from healthy and diseased adult articular cartilage. We summarize the state-of-the-art research and discuss its potential for clinical use in cartilage repair therapies.

## Results

**Fig. 1:**
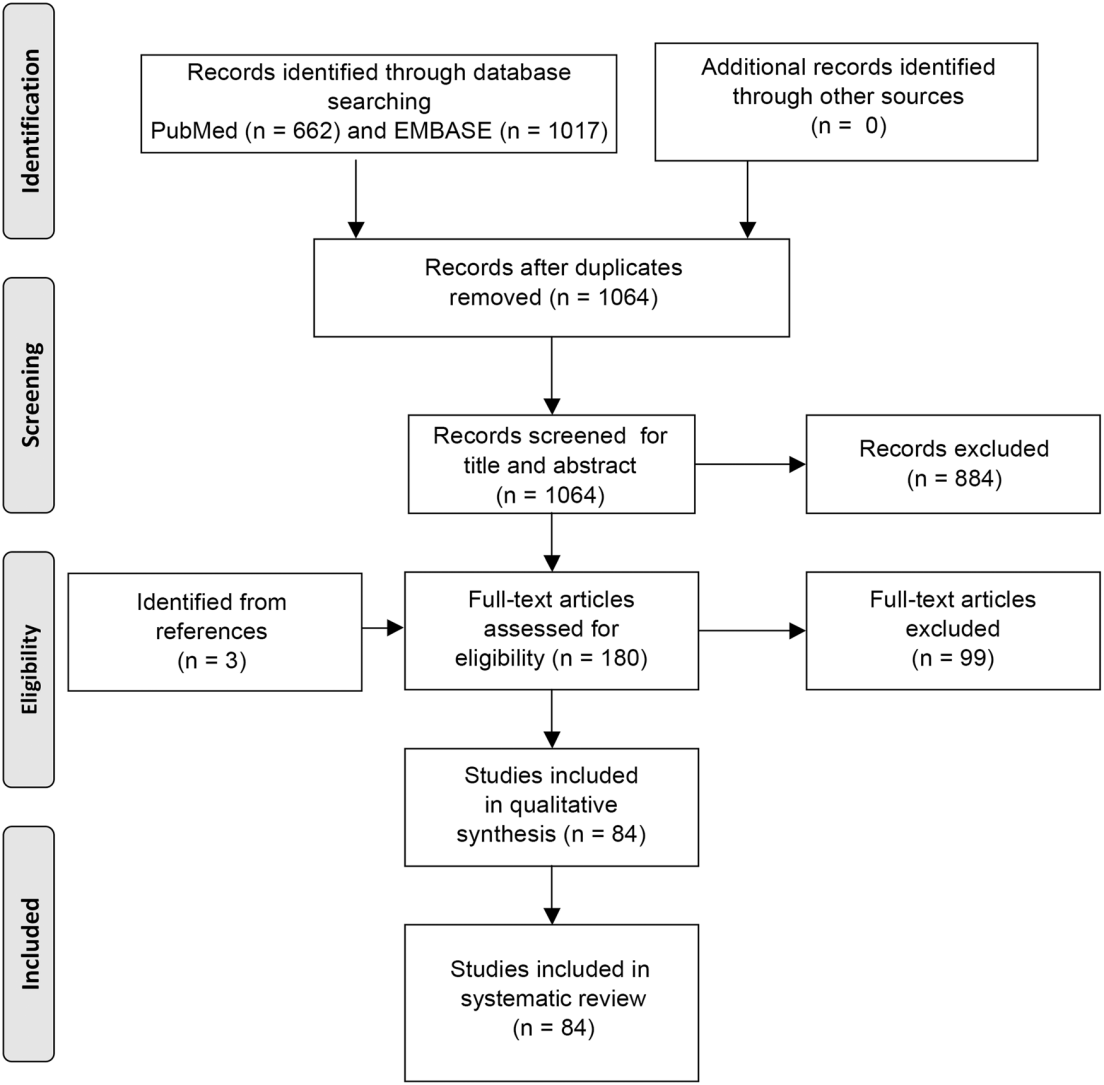
Flow diagram of the literature search. Preferred Reporting Items for Systematic Reviews and Meta-Analyses (PRISMA) workflow showing systematic selection process for studies.

The literature search yielded 1017 studies in EMBASE and 662 studies in PubMed. After duplicate removal, 1064 studies were identified. After title and abstract screening, the full text of 180 studies was screened. A total of 84 studies were then found eligible based on the inclusion and exclusion criteria (Fig. 1).

### Markers to identify ACPCs in vivo

The presence of ACPCs was first described by Dowthwaite et al. in 2004^16^. Enhanced expression of fibronectin and one of its key receptors, integrin-α5β1, was found in the superficial zone of bovine articular cartilage. Isolation of this fraction resulted in a population with high clonogenicity. As a unique marker or marker set is lacking, MSC or chondrocyte markers are mostly used for identification (Table 1). Classical MSC markers CD105^21–23^, CD166^24^, CD146^25^, VCAM^26^, or combinations including these markers^27,28^ have been used by others. In essence, this results in the identification of an MSC-like population in articular cartilage. Additional markers have been described to identify ACPCs in the tissue more specifically. Proteins involved in the Notch signaling pathway, like Notch-1, Notch-2, Delta, and Jagged^26,29^, or integrin-α5β1^21^, proteoglycan 4 (PRG4, or lubricin)^30^, and laminin^31^ are used. Alternative approaches to identify ACPCs in cartilage tissue have focussed on visual distinction by an elongated cell morphology of ACPCs in cartilage tissue samples^31,32^, cell clustering of ACPCs^33^, proliferation marker Ki-67^33,34^, and migration of ACPCs upon stimulation of the cartilage^35^.

**Table 1:** Identifying an ACPC population in articular cartilage.

	Species	Anatomical location of cartilage	Disease model/state	Method of progenitor identification in tissue	Outcomes
Tao et al.^22^	Murine	Knee	Unknown	CD105^+^ in the superficial layer	CD105^+^ cells in the superficial layer increased after induced OA and FN treatment. CD105^+^/CD166^+^ cells increased consistently
Tong et al.^34^	Rat	Knee	Unknown	Ki-67/BrdU labeling	Prevalence of ACPCs increases in OA. The highest frequency in the superficial layer. Inhibition of NF-κB pathway increased ACPCs in OA progression and lowered OARSI scores
Zhang et al.^21^	Rat	Hip	Unknown	CD105^+^/integrin-α5β1^+^ co-expression	CD105^+^/integrin-α5β1^+^ cells are activated by partial-thickness cartilage defects
Cai et al.^45^	Rat	Knee	ACLT-induced OA	CD44E^+^/CD90^+^ co-expression	Recovery of CD44E^+^/CD90^+^ cells in cartilage after ACLT and treatment with HA and magnoflorine
Walsh et al.^23^	Porcine	Knee	Unknown	Mechanical loading of immature, adolescent, and mature cartilage followed by surface marker expression, gene expression, and histology	Increased expression of CD105 and CD29 in immature cartilage; decreased expression of ACAN, Col-X and SOX9 in immature cartilage, increased expression of Col-I, Col-II in immature cartilage
Dowthwaite et al.^16^	Bovine	Articular cartilage (surface, middle, and deep zone)	Unknown	Expression of integrin-α5, integrin-β1, fibronectin, and Notch-1	All markers are mainly expressed in the superficial zone
Jang et al.^35^	Bovine	Stifle (tibial plateau)	Unknown	Calcein-AM/Ethidium homodimer staining of cells migrated into fibrin in partial- and full-thickness defects, treated with low-intensity pulsed ultrasound	More cells migrated in low-intensity pulsed treated defects. FAK activation increased in treated samples
Seol et al.^32^	Bovine and human	Stifle (bovine) and talus (human)	Healthy	Morphology	Increased number of elongated cells in impacted cartilage explants of both species
Ustunel et al.^29^	Human	Knee (intercondylar notch)	Healthy	Expression of Notch-1, Notch-2, Notch-3, Notch-4, Delta, Jagged-1, and Jagged-2	Notch-1 and Delta were abundantly expressed in the superficial zone
Grogan et al.^26^	Human	Knee	Healthy and OA	Expression of Notch-1, VCAM, and Stro-1	All markers show expression throughout all cartilage layers; expression in the superficial zone is increased
Pretzel et al.^24^	Human	Knee	Healthy and OA	Expression of CD166	High percentage (22%) of CD166^+^ cells. The highest prevalence in the superficial and middle zone
Su et al.^25^	Human	Knee (femoral condyles)	OA	Expression of CD146	CD146^+^ cells observed in OA cartilage and are smaller in size than CD146^−^ cells
Hoshiyama et al.^33^	Human	Knee (femoral condyles)	OA	Cell clustering; expression of Stro-1, FGF-2, Ki-67	More cell clustering and higher expression of all markers in cells adjacent to cartilage damage
Schminke et al.^31^	Human	Knee (lateral femoral condyles)	Healthy and OA	Morphology; expression of laminin-α1 and laminin-α5 in the pericellular matrix.	More laminins expressed in the pericellular matrix of cells with an elongated morphology
De Luca et al.^30^	Human	Hip (femoral head and neck)	Healthy and OA	Expression of PRG-4	Expression of PRG-4 shifts from the superficial layer (healthy cartilage) to deeper zones (OA cartilage)
Wang et al.^28^	Human	Knee (tibial plateau)	OA	CD271^+^ and CD105^+^ cell distribution in WORMS grade 1–2 versus 3–4 cartilage	Enhanced expression of CD105 and CD271 in the superficial zone of grade 3–4 cartilage

*ACLT* anterior cruciate ligament transection, *FAK* focal adhesion kinase, *FN* fibronectin, *HA* hyaluronic acid, *OA* osteoarthritis, *PRG-4* proteoglycan 4, *VCAM* vascular cell adhesion molecule.

### Methods for isolation of ACPCs from cartilage

A protocol for selective isolation of ACPC by differential adhesion to fibronectin (DAF) was established^16,17,29^, taking advantage of the enriched expression of the fibronectin receptor^16^ and the finding that isolation based on integrins resulted in selection for stem cells rather than transit-amplifying cells^36^. In two-thirds of the studies using DAF, this protocol is followed by isolation of colonies, that are subsequently formed by the cells that adhere (generally) in 20 min^13,14,17,18,22,29,37–43^. Six out of nineteen studies did not perform colony isolation and the complete pool of cells that adhered to fibronectin was isolated^34,44–48^.

Alternatively, ACPCs are sorted from the total cell population either via immunomagnetic separation or fluorescence-activated cell sorting (FACS). ACPCs were isolated by FACS based on co-expression of CD105 and CD166^15^, a marker combination that defines a subset of bone marrow-derived MSCs^49^ and was proposed to select for ACPCs. Another marker set used for cell sorting that resulted in an ACPC population is CD9^+^/CD90^+^/CD166^+^^50^.

Finally, cells migrating out of cartilage explants, whether or not the cartilage is stimulated in any way, hold progenitor characteristics such as multilineage differentiation potential and colony forming efficiency (CFE)^19,28,32,51–53^. These migratory cells were distinctly different from chondrocytes and osteoblasts^54^. To stimulate migration of cells, explants were stimulated by nerve growth factor (NGF)^52^, platelet lysate^19^, or migrating cells were isolated after partial digestion of the tissue by collagenase^28^. Cell migration could also be triggered by induction of injury^32,53^. Cells with progenitor characteristics migrated towards the site of cartilage injury and a role in the repair of adult cartilage upon damage was suggested by the authors^32^.

Nine studies did not report on any distinct method to isolate a population from the total cell population^7,30,55–61^. Five others performed an isolation step after one or two passages in culture^24,25,62–64^. It can therefore be questioned whether these are investigating a population that is different from what is generally referred to as chondrocytes, as most of the studies were also lacking a chondrocyte control group.

### In vitro characterization of ACPCs after isolation

Isolated ACPCs are characterized based on their proliferative potential, CFE, differentiation potential, and expression of markers that are also used for their isolation (Table 2). ACPCs could be maintained in culture for up to 30–60 population doublings^17,18,37–39^ and early-passage cells were able to form colonies in culture^7,16,18,19,28,32,39,46,52,65,66^. Moreover, human ACPCs were found to maintain telomere length and telomerase activity up to at least 20 population doublings^18,37^. However, ACPCs derived from OA cartilage contained a subpopulation of cells that have reduced proliferative potential and undergo early senescence when cultured in vitro^18^.

**Table 2:** Isolation and characterization of ACPCs.

Study	Species	Anatomical location of cartilage	Disease state	Isolation procedure of cells	Cell characterization		Compared to (cell type)
				**Differential adhesion to fibronectin**		**Marker expression**	
Tao et al.^22^	Murine	Knee	Unknown	DAF followed by colony isolation	Proliferation; migration; chondrogenic differentiation	CD34, CD45, CD105, CD166	Chondrocytes
Tong et al.^34^	Rat	Hip and knee	Unknown	DAF	Chondrogenic, osteogenic, and adipogenic differentiation	CD90, CD44, CD45, CD31, CD34	Chondrocytes; BM-MSCs
He et al.^44^	Rat	Knee	Unknown	DAF	Osteogenic and adipogenic differentiation	CD90, CD73, CD105, CD34, HLA-DR (after one passage)	—
Cai et al.^45^	Rat	Knee	OA (ACLT-induced)	DAF	Chondrogenic differentiation	CD44E/CD90 coexpression	—
Li et al.^46^	Rabbit	Knee (surface zone cartilage)	Healthy	DAF	CFE; chondrogenic, osteogenic, and adipogenic differentiation in alginate beads	—	Chondrocytes; IFP-stem cells
Dowthwaite et al.^16^	Bovine	Articular cartilage (surface, middle, and deep zone)	Unknown	DAF	Adhesion to FN; CFE	α5 and β1 integrin (immunolocalization)	—
Khan et al.^37^	Bovine	Juvenile metacarpophalangeal joint	Healthy	DAF followed by colony isolation	Population doublings; telomerase activity; telomere length; gene expression; chondrogenic differentiation	Sox-9; Notch-1; PCNA (all immunolocalization)	Full-depth and superficial zone chondrocytes
Marcus et al.^38^	Bovine	Metacarpophalangeal joint	Healthy	DAF followed by colony isolation	Population doublings	—	—
McCarthy et al.^14^	Equine	Metacarpal joint	Unknown	DAF followed by colony isolation	Chondrogenic, osteogenic, and adipogenic differentiation	Notch-1; Stro-1; CD90; CD166 (all immunolocalization)	BM-MSCs
Levato et al.^13^	Equine	Metacarpophalangeal joint	Healthy	DAF followed by colony isolation	Chondrogenic, osteogenic, and adipogenic differentiation	CD13; CD29; CD31; CD44; CD45; CD49d; CD73; CD90; CD105; CD106; CD146; CD166 (all gene expression)	BM-MSCs
Ustunel et al.^29^	Human	Knee (intercondylar notch)	Healthy (ACLT repair)	DAF followed by colony isolation	—	Notch-1; Notch-2; Notch-3; Notch-4; Delta; Jagged-1; Jagged-2 (all immunolocalization in colonies)	Chondrocytes
Williams et al.^17^	Human	Knee	Healthy	DAF followed by colony isolation	Population doublings; chondrogenic, osteogenic, and adipogenic differentiation; karyotyping; telomere length analysis; cell engraftment *in ovo*	Notch-1; CD90; Stro-1; Jagged-1; Delta-1 (all immunolocalization)	Full-depth chondrocytes
Nelson et al.^18^	Human	Knee (tibial plateau)	OA	DAF followed by colony isolation	CFE on FN; growth kinetics; chondrogenic, osteogenic, and adipogenic differentiation;	STRO-1	—
Fellows et al.^39^	Human	Knee (tibial plateau)	Healthy and OA	DAF followed by colony isolation	CFE on FN; growth kinetics; chondrogenic, osteogenic, and adipogenic differentiation; telomere length analysis	—	—
Shafiee et al.^40^	Human	Articular cartilage (not specified)	Unknown	DAF followed by colony isolation	Cell cycle analysis; karyotyping; proliferation; chondrogenic differentiation	CD166; CD133; CD106; CD105; CD90; CD73; CD 45; CD34; HLA-DR	Nasal septum-progenitors; BM-MSCs; AD-MSCs
Vinod et al.^41^	Human	Knee (superficial layer)	Healthy	DAF followed by colony isolation	Chondrogenic, osteogenic, and adipogenic differentiation	CD105; CD73; CD90; CD34; CD45; CD29; CD49e; CD151; CD166	Chondrocytes
Zhang et al.^47^	Murine and human	Knee	Healthy and OA	DAF	Chondrogenic, osteogenic, and adipogenic differentiation; proliferation; high-throughput RNA sequencing	PRDM16; NCAM-1; N-cadherin; CPNE-1; CTGF (protein expression). CD29; CD44; CD90; CD45; CD34	—
Kachroo et al.^48^	Human	Knee	OA	DAF	Gene expression	—	Non-DAF cells; fresh cartilage cells
Vinod et al.^42^	Human	Knee	Healthy and OA	DAF followed by colony isolation	Population doublings; chondrogenic, osteogenic, and adipogenic differentiation; gene expression	CD105, CD73, CD90, CD34, CD45, CD14, CD54, CD44, CD9, CD106, CD29, CD151, CD49e, CD166; CD146	Chondrocytes
Vinod et al.^43^	Human	Knee	Healthy and OA	DAF followed by colony isolation	Expression of immunogenic markers HLA-A2; HLA-B7; HLA-DR; CD80; CD86; CD14	—	Chondrocytes
				**Cell sorting**			
Karlsson et al.^65^	Bovine	Knee (femoral condyle)	Healthy	FACS for Notch-1 or cell size	CFE (in agarose); chondrogenic, osteogenic, and adipogenic differentiation	—	Notch-1− cells and larger/small cells
Alsalameh et al.^15^	Human	Knee (femoral condyle and tibial plateau)	Healthy and OA	Immunomagnetic cell separation for CD105^+^/CD166^+^	Chondrogenic, osteogenic, and adipogenic differentiation	CD105; CD166 (immunolocalization)	BM-MSCs
Fickert et al.^50^	Human	Knee	OA	FACS for CD9^+^/CD90^+^/CD166^+^	Chondrogenic, osteogenic, adipogenic differentiation	—	—
Pretzel et al.^24^	Human	Knee	OA	Immunomagnetic cell separation for CD166 (after one passage)	Chondrogenic, osteogenic, adipogenic differentiation	CD105; CD166 (immunolocalization)	—
Peng et al.^62^	Human	Hip (femoral head)	Healthy and OA	Immunomagnetic cell separation for CD105^+^/CD166^+^ (after one passage)	Chondrogenic differentiation	—	—
Su et al.^25^	Human	Knee (femoral condyles)	OA	FACS for CD146 (after one passage)	Chondrogenic, osteogenic, and adipogenic differentiation; gene expression; CFE (after three passages)	CD29; CD31; CD45; CD133; CD44; CD34; CD73; CD90; CD146; CD105; CD166; HLA-ABC; HLA-DR	Unsorted chondrocytes; AD-MSCs
Unguryte et al.^106^	Human	Knee	OA	FACS for ALDH activity	Gene expression	CD29; CD49a; CD49c; CD105; CD349; Notch1; CD54; CD55; CD56; CD63; CD47; CD140b; CD146; CD166	ALDH− and ALDH-diminished-expressing cells
Xia et al.^63^	Human	Knee (femoral condyles)	OA	FACS for CD105^+^/CD166^+^ (after two passages)	Cell proliferation; gene and miRNA expression; chondrogenic, osteogenic, and adipogenic differentiation	CD29; CD44; CD73; CD90; CD105; CD166; CD19; CD34; CD45; HLA-DR	—
Kachroo et al.^66^	Human	Knee	OA	FACS for CD49e^+^	CFE	CD49e; CD29	Fresh chondrocytes; CD49e^−^ cells
				**Migration from tissue**			
Joos et al.^51^	Human	Knee	OA	Outgrowth from cartilage tissue	Chondrogenic, osteogenic, and adipogenic differentiation; cell migration; chemotaxis	CD9; CD54; CD146; CD14; CD73; CD166; CD29; CD88; CD184; CD34; CD90; MSCA-1; CD44; CD105; Stro-1 (and quadruplicate combinations of these markers)	BM-MSCs
Jiang et al.^52^	Human	Knee (femoral condyles)	OA	Cell migration through a membrane stimulated by NGF	CFE; chondrogenic, osteogenic, and adipogenic differentiation	CD90; CD73; CD105; CD166; CD44; CD29; CD34; CD45	—
Carluccio et al.^19^	Human	Hip	OA	Outgrowth from cartilage tissue using platelet lysate	Growth kinetics; CFE; chondrogenic, osteogenic, and adipogenic differentiation; migration; chemotaxis; secretory profile; gene expression	Cyclin D1; α-tubulin (protein expression); CD44; CD166; HLA-ABC; HLA-DR; CD90; CD105; CD73; CD146; CD106; CD45; CD34; CD29	Chondrocytes
				**Enzymatic**			
Seol et al.^32^	Bovine	Stifle (tibial plateau)	Healthy	Trypsin treatment after injury	Migration; chemotaxis; chondrogenic, osteogenic, and adipogenic differentiation; RNA microarray; CFE	—	BM-MSCs; chondrocytes
Zhou et al.^53^	Bovine	Stifle (tibial plateau)	Healthy	Trypsin treatment after injury	Gene expression; chondrogenic differentiation	—	Chondrocytes; synoviocytes; synovial fluid cells
Wang et al.^28^	Human	Knee (tibial plateau)	OA	Collagenase treatment followed by outgrowth	Growth kinetics; CFE; chondrogenic, osteogenic, and adipogenic differentiation	CD29; CD31; CD44; CD45; CD73; CD90; CD105; CD166; CD271	—
				**Other isolation procedures**			
Hattori et al.^107^	Bovine	Stifle	Healthy	Hoechst 33342^-^	Chondrogenic differentiation	—	Hoechst 33342^+^ population
Yu et al.^72^	Bovine	Stifle (femoral condyle)	Healthy	Colony formation of single live cells	Chondrogenic, osteogenic, and adipogenic differentiation; gene expression; migration	—	Chondrocytes
Thornemo et al.^64^	Human	Knee	Healthy	Cluster growth in agarose (after one passage)	Chondrogenic, osteogenic, and adipogenic differentiation	—	Periosteal cells; BM-MSCs; fibroblasts
Grogan et al.^26^	Human	Knee	Healthy and OA	Hoechst 33342^-^	Chondrogenic, osteogenic, and adipogenic differentiation	—	Hoechst 33342^+^ population
				**No isolation procedure described**			
Barbero et al.^7^	Human	Knee (femoral condyle)	Healthy	—	CFE; proliferation rate; chondrogenic, osteogenic, and adipogenic differentiation	—	—
Tallheden et al.^55^	Human	Knee	Healthy	—	Chondrogenic, osteogenic, and adipogenic differentiation	—	BM-MSCs
Bernstein et al.^56^	Human	Knee	OA	—	Chondrogenic, osteogenic, and adipogenic differentiation	CD9; CD166; CD90; CD54; CD44; CD45; CD105; CD73; CD54 (quadruple combinations)	—
Salamon et al.^61^	Human	Knee	OA	—	Growth kinetics; adipogenic and osteogenic differentiation	CD29; CD44; CD105; CD166	AD-MSCs
Mantripragada et al.^58^	Human	Knee (femoral condyle)	OA	—	CFE; chondrogenic differentiation	—	—
Mantripragada et al.^57^	Human	Knee (femoral condyle)	OA	—	CFE; chondrogenic differentiation	—	—
De Luca et al.^30^	Human	Hip (femoral head and neck)	OA	—	CFE; chondrogenic, osteogenic, and adipogenic differentiation; immunomodulatory properties	CD14; CD34; CD44; CD45; CD71; CD105; CD166; CD90; CD73; CD151	BM-MSCs; AD-MSCs
Mantripragada et al.^59^	Human	Knee (femoral condyle)	OA	—	CFE; chondrogenic differentiation	—	BM-MSCs; IFP-cells; synovium-derived cells; periosteal cells
Mantripragada et al.^60^	Human	Knee (femoral condyle)	OA	—	CFE; chondrogenic differentiation	—	IFP-cells; synovium-derived cells; periosteal cells

*ACLT* anterior cruciate ligament transection, *AD* adipose tissue-derived, *ALDH* aldehyde dehydrogenase, *CFE* colony-forming efficiency, *DAF* differential adhesion to fibronectin, *FN* fibronectin, *IFP* infrapatellar fat pad, *NGF* nerve growth factor, *OA* osteoarthritis.

ACPCs could be differentiated into the chondrogenic, osteogenic, and adipogenic lineage, a feature that MSCs also possess^67^. There is one report of reduced osteogenic differentiation potential of ACPCs^42^, while 20% of the studies looking into multilineage potential found indications for reduced osteogenesis^12,13,19,20,50,68^.

Surface marker expression of ACPCs was in general similar to MSCs, with ACPCs being positive for CD90, CD105, CD73, and CD166, while negative for hematopoietic markers, highlighting the challenge to distinguish the two cell types^13,19,28,30,40–42,51,52,56,63^. Of note, about half of the studies mentioned here examine immunophenotype of cells in culture^13,19,22,28,30,34,40,42,44,47,51,56,61,66,69^, while cells tend to change their phenotype during in vitro expansion^70,71^. Moreover, investigating marker expression by gene expression or flow cytometry on the bulk populations makes it problematic to define whether these markers are co-expressed or not.

### In vitro comparison of ACPCs to other cell types with regard to surface marker expression

Cell surface marker expression and in vitro performance of ACPCs were directly compared to MSCs from various sources, like bone marrow^13–15,30,32,34,40,51,55,59,64^, adipose tissue^25,30,40^, and infrapatellar fat pad^46,59,60^. Other cell types compared are chondrocytes^17,19,22,25,29,32,34,37,41,42,46,48,53,65,66,72^ and other intra-articular cells, like synoviocytes^53,59,60^, synovial fluid cells^53^, and periosteal cells^59,60,64^ (Table 2).

A clear distinction between MSCs and ACPCs based on the expression of markers was only reported once, when equine ACPCs were compared to bone marrow-derived MSCs, an increase in gene expression for CD44 was found^13^. One-third of the studies directly compare ACPCs to chondrocytes, as these also reside in adult hyaline cartilage, and distinction of these cell types is crucial for isolation and application. The proliferation of ACPCs was faster than chondrocytes in one study^19^, but slower in a different report^42^. In addition, ACPCs were found to form more colonies compared to chondrocytes^32^. A distinction was made between chondrocytes and ACPCs based on high expression of CD90^17,25,34^, CD44^34^, CD105^46^, CD166^46^, Notch-1^17^, and HLA-ABC^25^ in ACPCs while culture-expanded chondrocytes showed little to no expression of these markers. Co-expression of CD44 and CD90 was found to distinguish between rat chondrocytes and ACPCs^34,45^. When ACPCs were sorted from the total pool of chondrocytes by CD49e-expression, a difference was found in the expression of CD29 in chondrocytes (50%) versus ACPCs (100%)^66^. When ACPCs were treated with platelet lysate, an increased expression of CD166 and decreased expression of CD106 compared to chondrocytes was found^19^.

### Differences between species

Identification of similarities and differences in ACPCs between species is challenging, due to the diversity of isolation procedures and variety of study objectives. Colony formation was identified in several human studies, as well as in the first report on bovine ACPCs. CFE in bovine cartilage cells was reported to be 0.6%^16^, while all other literature on non-human cells lacked this analysis. In human cells, consistency is found to some extent. CFE in healthy cartilage cells on fibronectin-coated dishes was 1.47%^39^, while this was almost double (2.8%) in OA cells in the same study. Others reported on CFEs of <0.1%^18^ and 0.66%^66^ of OA cells on fibronectin-coated dishes. When OA cells were seeded on uncoated culture plastic, a CFE of <0.01% was found^57–59^. The percentage of colony forming cells increased when cells were culture expanded. Passage one OA cells (isolation method not specified) had 18% CFE^30^ and the same passage cells that migrated from OA tissue in response to platelet lysate had 7.8% CFE^19^. Cells that migrated from OA tissue with NGF and were expanded for four passages had increased their CFE to 38.6%^52^. When CD105^+^/CD166^+^-sorted cells were quantified, CFEs of 3.5% (healthy) and 8% (OA) were found in one study^15^ and 15% (healthy) and 17% (OA) were found in another^24^. Of note, the latter used cells that were culture expanded for one passage. Overall, when comparing human ACPC studies, it seems that OA tissue contains more colony forming cells than healthy cartilage. Also, CFE increases after culture expansion, possibly as a consequence of culture-related changes in immunophenotype^71^.

### Differences between ACPCs from healthy and osteoarthritic cartilage

ACPCs have been identified in hyaline cartilage from different pathological states. Identification and characterization can contribute to our understanding of their role in homeostasis and disease, as well as their accessibility for clinical use.

In healthy articular cartilage, ACPCs most likely reside in the superficial zone, as Notch-1-expressing cells are found here^16^ and possess progenitor cell characteristics^17,26,29^. In addition, enhanced expression of fibronectin and one of its receptors, integrin-α5 and -β1, was found in the superficial zone^16^. As a direct consequence, most of the cells isolated via DAF originated from the superficial zone. The same group also showed that the CFE of surface zone cells is higher compared to deep zone cells^16^.

Upon damage of cartilage, ACPCs seem to migrate towards the site of injury^73^. Cells that migrated into the site of injury were found to possess progenitor-like characteristics^32^. An increase of CD271-expression was seen in ACPCs from increased OA severity^28^. Classical MSC markers CD105^21,22^, VCAM^26^, or combinations including these markers^27,28^ were all enhanced in OA cartilage or upon trauma. A shift of expression of PRG4^30^ from the superficial layer to deeper zones was seen in OA, whereas CD271- and CD105-positive cells shifted towards the superficial zone in OA^28^. In OA cartilage, cell clusters were observed which express ACPC-associated markers like Notch-1, Stro-1^27^, VCAM, FGF-2, and Ki-67^26,33^. These cells proliferated faster and produced more cartilaginous nodules in vitro compared to cells isolated from macroscopically healthy cartilage^33^. Contradictory, others found that ACPCs derived from healthy cartilage proliferated faster than OA-derived ACPCs^63^. Lastly, a high number of CD105/CD166-positive^15^ and CD146-positive cells^25^ was found in OA cartilage and these cells had multilineage potential. OA-derived cells also formed more colonies compared to cells from normal human cartilage^39^ and this increased with OA severity^28^.

### In vitro culture of ACPCs

Three studies made an attempt to optimize growth kinetics examining factors like seeding density, culture systems, and serum concentrations^74–76^. The authors reported on optimal expansion conditions when the medium was supplemented with fetal bovine serum (FBS) and transforming growth factor (TGF)-β1 at 40% and 1 ng/mL, respectively^74^. However, more recent studies have not used FBS concentrations that were as high as 40%. A passage length of 5 days was optimal for cell yield and the authors reported on reduced costs of expansion by 60%^76^. Furthermore, a method for expansion on microcarriers eliminated the need for a harvesting step and was thus suggested to prevent dedifferentiation^75^. A direct comparison of fibronectin versus laminin, another important cell adhesion molecule, for differential adhesion of ACPCs resulted in higher population doubling, increased gene expression of type II collagen, and increased osteogenic and adipogenic differentiation potential of laminin-selected ACPCs^69^. Likewise, expansion with platelet lysate compared to FBS showed more population doublings and increased expression of chondrogenic genes aggrecan and type II collagen, but at the same time expression of type X collagen was also increased^77^. Others found increased gene expression of aggrecan, type II collagen, and Sox9, as well as proteoglycan and type II collagen production of ACPCs by application of intermittent hydrostatic pressure^46^ or mechanical stimulation in a bioreactor system^78^, and inhibition of CFE by high glucose levels during growth culture^60^. Moreover, normoxic versus hypoxic conditions revealed greater production of glycosaminoglycans, low alkaline phosphatase expression, and weaker type I collagen staining in both conditions compared to MSCs^20^. In line, consistently low levels of type X collagen were expressed by ACPCs when normoxia and hypoxia were compared^68^. A reduction of oxygen tension during culture is also known to delay chondrocyte aging and improve their chondrogenic potential^79,80^.

In brief, the optimization of culture conditions for ACPCs has been investigated extensively. There are no uniform protocols for expansion and optimal differentiation for cartilage formation. Consensus on these matters would aid in comparing outcomes of studies in the future.

Upon ex vivo injury of bovine cartilage, migratory cells with progenitor characteristics were found^32,35^. Additional research showed that the phagocytic capacity of these ACPCs was higher compared to chondrocytes and comparable to synoviocytes and macrophages, suggesting a macrophage-like role for ACPCs in cartilage injury^81^. After treating ACPCs with supernatant from injured explants, proliferation, migration, and expression of immunomodulatory mediators were enhanced, while chondrogenic capacity was impaired^27^.

Stimulation of chondrogenesis in ACPCs was successful by inhibition of the nuclear factor-κB pathway, the major signaling pathway involved in OA^82^. Inhibition of this pathway was achieved by an inhibiting peptide^34^ and magnoflorine^45^, both resulting in increased chondrogenesis. Interleukin-1Β and tumor necrosis factor-α, inflammatory factors involved in OA, were reported to inhibit migration of ACPCs^51^. Similarly, β-Catenin and NGF are elevated in OA^83,84^. Inhibition of the Wnt/β-Catenin pathway promoted proliferation and differentiation^62^, while NGF failed to stimulate chondrogenesis in ACPCs^52^. The specific role of these compounds in OA remains to be investigated.

Alternatively, chondrogenesis could be triggered by the direct activation of chondrogenic pathways. Combined mechanical stimulation and shear stress-induced chondrogenesis through an increase in endogenously produced TGF-β1, while overexpression of BMP2 reduced chondrogenesis^78^. Also, BMP9 was a potent stimulator of chondrogenesis^85^. Direct treatment of ACPCs with extracellular matrix components Link protein N-terminal peptide^44^ or nidogen-2^31^ increased expression of chondrogenic genes. The proliferation of ACPCs was promoted by kartogenin^86^, a small molecule that induces chondrogenic differentiation of MSCs. Finally, sex hormones estrogen and testosterone influenced human ACPC performance^87^.

To summarize, initial results indicate that ACPCs respond to injury and chondrogenesis can be induced in vitro, which could make the cells interesting as therapeutic targets. These findings could be used to provoke neo-cartilage formation or inhibit inflammation in OA.

### Application and translation of progenitors

The potential of ACPCs for tissue engineering, biofabrication, and clinical application has been investigated widely (Table 3). Biofabrication allows for the production of constructs consisting of (bio)materials, bioactive cues, and/or cells, with a detailed predefined architecture^88^. The extensive proliferative potential of ACPCs combined with their chondrogenic capacity make these cells good candidates to use in tissue engineering and biofabrication approaches to repair or regenerate articular cartilage.

**Table 3:** Application and translation of ACPCs.

Study	Species	Anatomical location of cartilage	Disease state	Isolation procedure of cells	Other cell types compared	Application(s)	Outcomes
In vitro studies							
He et al.^44^	Rat	Knee	Unknown	DAF	—	Effect of LLP on cytotoxicity, chondrogenesis, proliferation, migration, chemotaxis, gene, and protein expression	No difference in cytotoxicity, proliferation; migration, chemotaxis, and chondrogenesis were increased by LLP; Sox9, Col-II, and Acan gene expression increased with LLP
Melero-Martin et al.^74^	Bovine	Juvenile metatarsophalangeal joint (superficial zone)	Healthy	DAF	—	Effect of cryopreservation on proliferation, viability, and chondrogenesis. Comparison between media and FBS, TGF-β1, and FGF concentrations	Cell density increased 53-fold with optimized FBS concentration up to 40% and feeding rate above 10 μL/cm^2^/h. Cell density increased 33-fold when media was supplemented with 1 ng/mL TGF-β1 and 40% FBS. Chondrogenic differentiation potential was maintained
Melero-Martin et al.^76^	Bovine	Juvenile metatarsophalangeal joint (superficial zone)	Healthy	DAF	—	Effect of seeding density, passage number, and feeding strategy on cell density	Optimal growth kinetics at 10^4^ cells/cm^2^ seeding density and 73 h passage length. However, looking at costs of expansion, a longer culture time was preferred
Melero-Martin et al.^75^	Bovine	Juvenile metatarsophalangeal joint (superficial zone)	Healthy	DAF	—	Growth kinetics 2D versus 3D microcarriers and differentiation potential afterward	Expansion slower than in 2D, but upscaling possible and chondrogenic differentiation potential maintained; bead-to-bead migration possible (subcultivation without harvesting)
Seol et al.^32^	Bovine	Stifle (tibial plateau)	Healthy	Enzymatic: Trypsin treatment after injury	BM-MSCs; chondrocytes	Migration of GFP-labeled grafted ACPCs into an impacted area on osteochondral explant	The number of labeled cells in the impact site increased drastically from 2 to 12 days (no quantification)
Jang et al.^35^	Bovine	Stifle (tibial plateau)	Unknown	Enzymatic: Trypsin treatment after injury	—	Cell migration under influence of low-intensity pulsed ultrasound	Low-intensity pulsed ultrasound stimulated migration of isolated ACPCs into scratch
Zhou et al.^81^	Bovine	Stifle (tibial plateau)	Healthy	Enzymatic: Trypsin treatment after injury	Chondrocytes; synoviocytes	Phagocytic capacity	ACPCs internalized more cell-debris than chondrocytes; similar to synoviocytes and (murine cell line) macrophages; ACPCs overexpressed markers associated with phagocytosis and internalized more FN fragments than chondrocytes
Morgan et al.^85^	Bovine	Immature metacarpophalangeal joint	Healthy	DAF followed by colony isolation	—	Determination of optimal potent chondrogenic factors	BMP9 increased aggrecan and Col-II gene expression, low Col-X expression, more anisotropic collagen fibril deposition
Koelling et al.^87^	Human	Knee	OA	Outgrowth from cartilage tissue	—	Effect of sex hormones on the regenerative potential	Sex hormones influence the regenerative potential of progenitor cells
Joos et al.^51^	Human	Knee	OA	Outgrowth from cartilage tissue	—	Cell migration under the influence of IL-1Β and TNF-α	Cell migration was inhibited by both IL-1Β and TNF-α
Peng et al.^62^	Human	Hip (femoral head)	Healthy and OA	Immunomagnetic cell separation for CD105^+^/CD166^+^ (after one passage)	—	Effect of Wnt-signaling on chondrogenic differentiation	Inhibition of Wnt/β-catenin promoted proliferation and differentiation
Jiang et al.^52^	Human	Knee (femoral condyles)	OA	Cell migration through Transwell stimulated by NGF	—	Influence of NGF on chondrogenesis	Chondrogenesis was not stimulated by NGF
Schminke et al.^31^	Human	Knee (lateral femoral condyles)	OA	Outgrowth from cartilage tissue	—	Effect of laminin or nidogen-2 on gene expression; Nidogen-2 siRNA applied	SOX9 and ACAN increased by nidogen-2. COL2A1 increased and COL1A1 decreased by laminin. ACPCs expressed more Nidogen-2 compared to both chondrocyte types. siRNA knockdown of nidogen-2 caused increased RUNX2 and decreased SOX9 protein expression
Anderson et al.^68^	Human	Knee (femoral condyles)	Healthy	DAF followed by colony isolation	—	Response to normoxia and hypoxia in pellets	Variation in intrinsic chondrogenicity between clones. ACPCs demonstrate a consistently low COLX gene and protein expression in physoxia
Nguyen et al.^108^	Human	Hip and knee	OA	—	—	Expansion with FBS versus PL	PL induces re-entry of the cell cycle, stimulates proliferation; PL-expanded cells better at producing cartilage; PL induces cell outgrowth from cartilage pieces
Anderson et al.^109^	Human	Knee (femoral condyles)	Healthy	DAF followed by colony isolation	—	Tissue self-assembly on membranes	Oriented cartilaginous tissue self-assembly by ACPCs on FN membranes. Higher GAG and collagen when compared to chondrocytes; surface lubricin was lower in ACPCs
Riegger et al.^27^	Human	Knee (femoral condyles)	OA	Outgrowth from cartilage tissue	—	Treatment of cells with explant supernatants (impacted or treated with compounds); chondrogenic capacities; gene expression for pro- and anti-inflammatory factors	Enhanced proliferation, migration, and expression of immunomodulatory mediators. Chondrogenic capacity was impaired
Vinod et al.^110^	Human	Knee (superficial layer)	Healthy	DAF followed by colony isolation	—	Micron-sized superparamagnetic iron oxide (M-SPIO) particle uptake and function thereafter	Viability, cell-markers, and chondrogenesis reduced with increasing concentration M-SPIO; osteogenic and adipogenic differentiation were unchanged
Vinod et al.^41^	Human	Knee (superficial layer)	Healthy	DAF followed by colony isolation	Chondrocytes	Cocultures of ACPCs and chondrocytes in different ratios	No difference in surface marker expression, gene expression, or growth kinetics
Vinod et al.^111^	Human	Knee (superficial layer)	OA	DAF followed by colony isolation	—	Trilineage differentiation and viability of ACPCs in PRP clots	Maintained differentiation potential and viability in PRP clots
Kachroo et al.^77^	Human	Knee	OA	DAF followed by colony isolation	—	Expansion of ACPCs with 10% FBS versus 10% hPL	hPL-expanded ACPCs had more population doublings, higher expression of CD146, and increased gene expression of COL2A1, ACAN, COL1A1, COL10A1
Mantripragada et al.^60^	Human	Knee (femoral condyle)	OA	—		Growth of ACPCs in high glucose (25 mM) and low glucose (5 mM)	CFE was inhibited by glucose
Vinod et al.^69^	Human	Knee	OA	DAF or differential adhesion to laminin followed by colony isolation	—	Comparison of fibronectin versus laminin adhesion assay for ACPC isolation	Higher population doublings in laminin-selected ACPCs; No difference in expression of CD105, CD73, CD90, CD34, CD45, HLA-DR, CD146, CD166, CD49e, and CD29; increased expression of COL2A1 in laminin-selected ACPCs; Increased osteogenic and adipogenic differentiation
Wang et al.^28^	Human	Knee (tibial plateau)	OA	Collagenase treatment followed by outgrowth	—	Differentiation; gene expression; migration (upon treatment with OA SF); comparison of grade 1–2 and 3–4 ACPCs	Grade 3–4 ACPCs showed enhanced migratory, osteogenic, and adipogenic potential; decreased chondrogenic potential
Vinod et al.^112^	Human	Knee	Healthy	DAF followed by colony isolation	—	Chondrogenesis under influence of a pulsed electromagnetic field	No difference between TGF-β2-treated ACPC pellets and pellets treated with a pulsed electromagnetic field
Tissue engineering studies							
Li et al.^46^	Rabbit	Knee (surface zone cartilage)	Healthy	DAF	Chondrocytes; IFP-stem cells	Effect of intermittent hydrostatic pressure on ACPCs in alginate beads	Increase in migration, proliferation, GAG production, Col-II production, chondrogenic gene expression under influence of intermittent hydrostatic pressure
Schmidt et al.^20^	Equine	Metacarpophalangeal joint	Healthy	DAF followed by colony isolation	BM-MSCs	3D culture in agarose in normoxic versus hypoxic conditions. Monocultures of ACPCs and MSCs, and zonal construct of ACPC/MSC	Higher production of glycosaminoglycans by ACPCs in normoxia and hypoxia. Weaker type I collagen staining in ACPC constructs, low ALP expression
Neumann et al.^78^	Human	Knee (tibial plateau)	Healthy	DAF followed by colony isolation	—	BMP-2 overexpression through adenovirus; Scaffold culture loaded versus unloaded	Loading induced chondrogenesis; chondrogenesis reduced by BMP2 overexpression
Shafiee et al.^40^	Human	Articular cartilage (not specified)	Unknown	DAF followed by colony isolation	Nasal septum progenitors (NSPs); BM-MSCs; AD-MSCs	Chondrogenesis and proliferation on nanofibrous scaffolds (PCL/PLLA).	Expression of SOX9 and ACAN higher in NSPs compared to ACPCs; COL1 and COL2 lower in ACPCs compared to NSP and AD-MSC
Biofabrication studies							
Levato et al.^13^	Equine	Metacarpophalangeal joint	Healthy	DAF followed by colony isolation	Chondrocytes; BM-MSCs	Cartilage formation in (layered) casted GelMA hydrogel constructs; cartilage formation in layered bioprinted cartilage construct (MSCs in middle/deep layer, ACPCs in superficial layer)	ACPCs produced a higher amount and better-quality neo-cartilage matrix compared to chondrocytes, but not MSCs; Interplay of ACPCs with chondrocytes and MSCs supported neo-cartilage synthesis in layered co-cultures
Lim et al.^94^	Equine	Metacarpophalangeal joint	Healthy	DAF followed by colony isolation	—	Chondrogenic differentiation in DLP-printed bio-resin constructs	DLP-printed bio-resin supported chondrogenic differentiation of ACPCs
Mouser et al.^89^	Equine	Metacarpophalangeal joint	Healthy	DAF followed by colony isolation	—	Encapsulation in GelMA/gellan/HAMA hydrogels and 3D (zonal) bioprinting	Successful chondrogenic differentiation in hydrogel
Bernal et al.^95^	Equine	Metacarpophalangeal joint	Healthy	DAF followed by colony isolation	—	Fibrocartilage formation in volumetric bioprinted meniscus-shaped constructs	GAG, type I and II collagen production; increased compressive modulus after chondrogenic culture
Diloksumpan et al.^90^	Equine	Metacarpophalangeal joint	Healthy	DAF followed by colony isolation	—	Encapsulation in GelMA in a biofabricated osteochondral plug	ACPCs produce cartilage matrix and differentiation of ACPCs was not hampered by the presence of a bone scaffold
Mancini et al.^91^	Equine	Metacarpophalangeal joint	Healthy	DAF followed by colony isolation	BM-MSCs	Encapsulation in hyaluronic acid/poly(glycidol) hybrid hydrogel in a layered biofabricated osteochondral plug in an equine model	No difference in histological scoring. Repair tissue was stiffer in ACPC/MSC zonal constructs compared to constructs containing MSCs only
Peiffer et al.^92^	Equine	Metacarpophalangeal joint	Healthy	DAF followed by colony isolation	—	Encapsulation of ACPCs in hydrogel reinforced with a melt electrowritten scaffold printed on curvature	Cartilage-like tissue formation throughout the construct with high shape fidelity
Piluso et al.^93^	Equine	Metacarpophalangeal joint	Healthy	DAF followed by colony isolation	BM-MSCs; DPSCs	Cytocompatibility of riboflavin and sodium persulfate; cytocompatibility in silk fibroin hydrogel	Riboflavin did not affect viability, sodium persulfate decreased viability after three hours in high concentration. ACPCs in hydrogel maintained viability over 28 days of culture
In vitro and in vivo studies							
Tao et al.^22^	Murine	Knee	Unknown	DAF followed by colony isolation	Chondrocytes	Effect of FN on proliferation, migration, and chondrogenesis. Effect of FN in early in vivo OA model	Increased proliferation, migration, and Col-II and Aggrecan expression by FN. Inhibited by integrin-α5β1 inhibitor. FN promoted cartilage repair in vivo and increased CD105^+^ and CD166^+^ cells
Wang et al.^113^	Murine	Joint (not further specified)	Unknown	DAF followed by colony isolation	—	EVs from MRL/MpJ super-healer mice-ACPCs were used for intra-articular injection in an OA model and for chondrocyte migration and proliferation	Super-healer mice ACPC-EVs could ameliorate OA severity in vivo and improve chondrocyte function in vitro
Tong et al.^34^	Rat	Hip and knee	Unknown	DAF	Chondrocytes; BM-MSCs	Chondrogenesis under influence of IL-1Β and NF-κB pathway inhibitor	NF-κB pathway inhibitor was successful in rescuing ACPC chondrogenesis
Cai et al.^45^	Rat	Knee	OA (ACLT-induced)	DAF	—	Chondrogenesis and migration under influence of magnoflorine	Chondrogenesis and migration were stimulated by magnoflorine
Liu et al.^86^	Rat	Knee	Unknown	—	—	Effect of kartogenin on ACPCs	Kartogenin promoted proliferation; increased percentage of G2-M stage cells, increased gene expression of IL-6 and Gp130; phosphorylation of Stat3 enhanced. In vivo destabilization of the medial meniscus: increased cartilage thickness after kartogenin injection; upregulation of Stat3 phosphorylation; enhanced distribution of CD44^+^/CD105^+^ cells
Williams et al.^17^	Caprine	Knee	Healthy	DAF followed by colony isolation	Full-depth chondrocytes	Caprine in vivo cartilage defect filling with cell-seeded type I/III collagen membrane	Good integration with surrounding cartilage. No difference between full-depth chondrocytes and ACPCs
Tallheden et al.^55^	Human	Knee	Healthy	—	BM-MSCs	In vivo osteochondrogenic assay in SCID mice	Cartilage matrix formation in the chondrocyte group compared to bone matrix formation in the MSC group
Carluccio et al.^19^	Human	Hip	OA	Outgrowth from cartilage tissue using platelet lysate	Chondrocytes	In vivo ectopic chondrogenesis and osteogenesis (pellet and biomaterials)	ACPCs (PL expanded) provided a better option than chondrocytes for stable cartilage regeneration
In vivo studies							
Marcus et al.^38^	Bovine	Metacarpophalangeal joint	Healthy	DAF followed by colony isolation	—	Intramuscular injection in SCID mice	ACPCs were able to survive but failed to produce cartilage matrix (while chondrocytes did)
Frisbie et al.^96^	Equine	Trochlear ridge of the femur (superficial zone)	Healthy	DAF followed by colony isolation	—	In vivo chondral defect filling in autologous fibrin, comparison of autologous and allogeneic cells	Autologous cells provide a benefit in outcomes in terms of pain, synovial effusion, range of motion, radiographs, and histology. No apparent benefit of allogeneic cells
In human studies							
Jiang et al.^12^	Human	Knee (femoral condyle)	OA	—	—	MACT procedure using ACPCs	Significant clinical improvement based on IKDC and Lysholm scores; full coverage of defect site after one year; hyaline-like cartilage architecture

*ACLT* anterior cruciate ligament transection, *AD* adipose tissue-derived, *ALP* alkaline phosphatase, *CFE* colony-forming efficiency, *DAF* differential adhesion to fibronectin, *DLP* digital light processing, *EV* extracellular vesicle, *FBS* fetal bovine serum, *FN* fibronectin, *GAG* glycosaminoglycan, *IFP* infrapatellar fat pad, *IKDC* International Knee Documentation Committee, *LLP* Link protein N-terminal peptide, *MACT* matrix-assisted autologous chondrocyte transplantation, *NGF* nerve growth factor, *NSP* nasal septum progenitor, *OA* osteoarthritis, *PCL* polycaprolactone, *PL* platelet lysate, *PLLA* polycaprolactone/polylactic acid, *PRP* platelet-rich plasma, *SCID* severe combined immunodeficient mice.

Under the influence of intermittent hydrostatic pressure, the performance of rabbit ACPCs embedded in alginate was enhanced significantly. These cultures were pretreated for 1 week with a TGF-β3-containing medium but did not receive any exogenous growth factors thereafter. After two and four weeks, glycosaminoglycan, collagen, and DNA content were significantly higher than groups not treated with intermittent hydrostatic pressure^46^. Two studies investigating equine ACPC performance in hydrogels both reported good outcomes. When the cells were embedded in gelatin methacrolyl (gelMA) hydrogel cultured in a chondrogenic medium, mainly a difference was found in the expression of zonal markers compared to bone marrow-derived MSCs. Expression of PRG4 was increased in ACPC-loaded gels, while type X collagen expression was decreased compared to MSCs^13^. Furthermore, when equine ACPCs were embedded in gelMA/gellan and gelMA/gellan/HAMA hydrogels and cultured in a chondrogenic medium, these produced more glycosaminoglycans and type II collagen than chondrocytes, whereas the performance of MSCs in the same gels was comparable to ACPCs^89^. Similar to hydrogels, printed scaffolds have also been successfully seeded with ACPCs. Human ACPCs seeded on fibrin-polyurethane composite scaffolds were responsive to mechanical stimulation. The cells produced more glycosaminoglycans and aggrecan gene expression was increased without the addition of exogenous growth factors^78^. Furthermore, human ACPCs could also be seeded onto polycaprolactone/polylactic electrospun nanofibrous scaffolds where the cells attached and spread over the fibers. Further research has to shed light on the chondrogenic performance of the cells in this specific setting^40^.

Besides tissue engineering, ACPCs were successfully used in several biofabrication techniques. It was shown that equine ACPCs have the potential to be bioprinted and while exact mechanisms remain to be elucidated, an interplay between MSCs, ACPCs, and chondrocytes was found to be important for neo-cartilage synthesis^13^. The same cells were also successfully used for encapsulation in various hydrogels^90–93^ in combination with biofabrication techniques like extrusion-based bioprinting^13,89^, digital light processing^94^, and volumetric bioprinting^95^, while maintaining cell viability. While these are only the first indications to use ACPCs with various techniques, additional research is necessary to assess chondrogenic performance of the cells in these settings. Nevertheless, initial results are promising to move forward with this cell population.

Several attempts were made to take the next steps in the application of ACPCs for in vivo cartilage formation and repair. These are important to translate in vitro findings and define the potential of ACPCs for the clinic.

When DAF-selected ACPCs were applied in a caprine model for cartilage defect filling using a cell-seeded type I/III collagen membrane (Chondro-Gide®), ACPC-seeded scaffolds showed good lateral integration with the surrounding tissue and type II collagen-positive repair tissue. However, no difference was found between chondrocyte- or ACPC-treated defects^17^. In the same study, engraftment into the growth plate of developing chick hind limbs of isolated and culture-expanded ACPCs was shown. Contradictory, DAF-selected bovine ACPCs that were injected intramuscularly in immune-deficient mice failed to produce cartilage matrix^38^. In an equine model, DAF-selected ACPCs were applied in a layered biofabricated osteochondral plug and showed good integration with the native cartilage, but the repair tissue contained mainly type I collagen^91^. When autologous and allogeneic ACPCs were directly compared in an equine cartilage defect model, an advantage of autologous over allogeneic cells was seen in histological outcomes^96^.

When human ACPCs were used in immune-deficient mice, the cells were successful in the production of cartilage matrix, whereas MSCs produced mainly bone^55^. The cells in this study were not isolated using any distinct method for ACPC isolation but were 2D expanded in low density with low glucose. Furthermore, migratory human ACPCs expanded using platelet lysate outperformed chondrocytes in an in vivo *e*ctopic chondrogenesis assay in athymic mice^19^.

Finally, an attempt was made to proceed to human application, by using ACPCs to replace chondrocytes for matrix-assisted autologous chondrocyte transplantation, similar to the caprine study mentioned earlier. The pilot study with 15 patients^12^ reported on repair tissue rich in type II collagen and proteoglycans and without types I and X collagen. Furthermore, IKDC and Lysholm questionnaire scores improved significantly. However, there was no direct comparison between ACPCs and expanded chondrocytes in this study.

While the discussed studies provide initial evidence of in vivo chondrogenic potential of these cells, further investigation is essential to ascertain promise cartilage repair and clinical translatability.

## Discussion

**Fig. 2:**
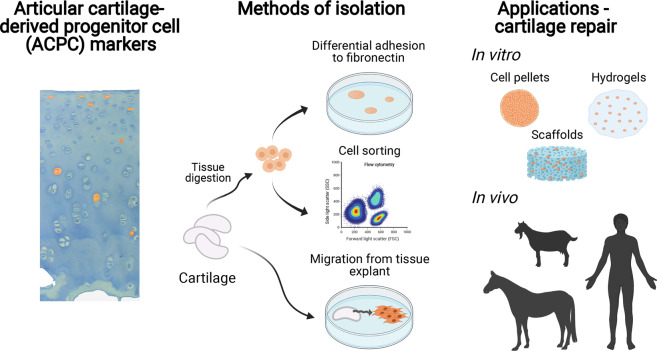
Isolation, characterization, and application of articular cartilage-derived progenitor cells. Schematic overview of the identification of articular cartilage-derived progenitor cells (ACPCs) in cartilage, isolation methods, and applications of ACPCs. Created with BioRender.com.

With this review, we aimed to systemically evaluate the available literature on adult ACPCs and their use for cartilage tissue engineering and repair therapies. We are the first to provide a thorough overview of research from the last two decades that demonstrates the presence of a progenitor cell population residing in adult hyaline cartilage (Fig. 2). Although a great effort was made to study the identity and applications of ACPCs, many uncertainties remain. As a result of differences in isolation protocols, characterization, and culture expansion, most cell populations discussed in the literature are likely to be heterogeneous populations and difficult to compare between laboratories. This stresses the need for this systematic review to expose certain inconsistencies and arrive at a shared definition of ACPCs.

The reviewed literature employs a wide variety of procedures for the isolation and characterization of ACPCs. Broadly speaking, three main methods for ACPC isolation are described. The method using DAF, used in 42% of the investigated studies, is based on the enriched expression of integrin-α5β1, as first described by Dowthwaite et al.^16^. The other two main methods are based on the expression of (a combination of) cell surface markers (19%)^15,50^ or migratory capacity (6%)^51^. Most populations isolated through these methods employed multilineage potential, responded to acute injury or mobilized during OA, and were able to produce hyaline cartilage extracellular matrix in vitro or in vivo.

The heterogeneity in isolation and characterization creates discrepancies between donors and laboratories. Direct comparisons of ACPC populations isolated through different procedures are lacking and would aid to improve our understanding of the populations. The identification of a unique cell marker would facilitate extensive and coordinated research into the cell type. This could pave the way towards clinical application or cell targeting to promote cartilage regeneration in OA. Recently, Gdf5-expressing cells in developing joints were identified to contribute to joint cell lineages^97^. Co-expression of Lgr5 and Col22a1 was identified as an important lineage marker towards juvenile articular chondrocytes in the developing mouse joint^98^. Additionally, single-cell RNA sequencing has revealed several novel markers that are potentially specific for ACPCs in human OA cartilage^99^.

The available literature suggests that ACPCs resemble MSCs^67^ in vitro based on surface marker expression and multilineage potential. The comparison to MSCs is often made due to the fact that MSCs (derived from various tissues) are a useful cell type for clinical application and are currently applied^9^. As the general view on the origin and role of MSCs is changing^100^, characterization of ACPCs based on MSC features might not be the way to go and other routes should be investigated. More recent work has shed light on the cellular basis of bone and cartilage formation by identifying skeletal stem cells in mice and humans^101,102^, a cell type that might be closer related to (the origin of) ACPCs in adult hyaline cartilage. Although the comparison to clinically used chondrocytes is relevant, research into similarities between ACPCs and skeletal stem cells or more downstream progenitor cells is lacking and finding resembling features would contribute to knowledge about the origin and identity of ACPCs.

Establishing the role of ACPCs in cartilage development and homeostasis, as well as their response upon injury or in OA would provide additional insights into their physiological function in mature cartilage. Regeneration in the early stages of OA could be stimulated or progression of the disease halted. Several studies discussed here suggested that ACPCs have a possible role in immunomodulation, based on their capacity to migrate upon injury^32,35^, excretion of inflammatory mediators^27^ and phagocytic capacities^81^. Others found a higher prevalence of these cells upon cartilage damage and OA^21,22,26–28,30,32–35^. Of note, these are all in vitro indications for which in vivo validation is essential. During OA, cell density and clustering in cartilage increases^103^, for which ACPCs might partly be responsible. On the other hand, there is contradicting evidence that Prg4-expressing cells from the synovium migrate into sites of acute cartilage injury and contribute to cartilage repair^97^. In order to expand the application of ACPCs to OA besides repair of chondral defects alone, immunomodulatory properties should be demonstrated in vivo as is known for MSCs^104^.

The described ACPC populations generally surpassed other cell types in proliferative potential and producing cartilage extracellular matrix in vitro^19,20,25,42,46^. In addition, most studies implanting animal-derived ACPCs in vivo confirmed their chondrogenic potential^17,38,91^, and even two studies using human cells reported on successful neo-cartilage formation^19,55^. As isolation methods do not seem to be associated with in vivo outcomes of cell performance and tissue formation, the challenge remains to compare findings between studies. Furthermore, differences between species and pathological states could influence cell performance. Donor age might play an important role, although none of the studies investigated this specifically. Nevertheless, the cells’ potency of prolonged in vitro expansion^17,19,37^ combined with limited tendency towards hypertrophic differentiation^13,14,19,68^ and their ability to form neo-cartilage can make ACPCs an appropriate cell type for repair of focal chondral defects.

Despite the great deal of research that has been done on ACPCs, certain actions need to take place in order to close the gaps and reach consensus between researchers and laboratories. As noted before, isolation based on a unique marker is crucial to ascertain similarity in cell population between laboratories. Comparison of culture media and additives for ACPCs in a recent systematic review^105^ highlights the importance of consistency to align research. As ACPCs currently have no discrete set of cell surface markers that can be used to isolate the cells from tissue, the question remains whether ACPCs are a distinct cell type or it refer to a heterogeneous mix of many cell types. The establishment of a cell marker and consistency in isolation and culture protocols ascertain comparability between populations. Another limitation might be the availability of tissue for cell isolation. Cartilage from OA patients is generally more accessible than healthy cartilage, as it is redundant after knee replacement surgery. A direct comparison of ACPC populations of healthy and OA cartilage would shed light on differences in performance. In the same view, investigation of allogeneic use of ACPCs is valuable, as this would greatly improve the potential of application by availability, reduction of costs, and preselection of chondrogenic cells.

The current systematic review is limited by the restriction to cell populations that are isolated from adult hyaline cartilage. The comparison and relation to cell types in the developing joint are lacking and would contribute to our further understanding of the origin of the populations discussed here and their role in joint development and homeostasis. However, the current review and discussed literature are predominantly directed at clinical translation as opposed to etiology or the role of a cartilage progenitor cell in development.

Arriving at a shared definition of a homogenous cell population that can be isolated and characterized in a comparable manner is crucial. This work could be used as a basis for research groups and clinicians to harmonize study protocols and characterization. First, studies should report on the origin of the cell in terms of species, anatomical location of the hyaline cartilage, and disease state. Second, the method of isolation should be described in detail and preferably identical to one of the established protocols. Finally, the phenotype of the isolated populations should be examined directly following isolation and culture media (and additives) as well as expansion time and/or passage number should be reported and synchronized.

To conclude, the available literature indicates that a cell population with progenitor-like characteristics resides in adult hyaline cartilage, which has extensive chondrogenic and proliferative potential. These features highlight the suitability of ACPCs as a cell source for focal chondral repair. In addition, it is crucial to investigate the role of ACPCs in development and disease, in order to determine their potential to slow down or reverse OA. If the current challenges can be overcome and consensus can be reached on this population, ACPCs hold great potential as a cell type for tissue engineering and for the repair of cartilage damage in both focal cartilage injury and OA.

## Methods

### Literature search

A systematic search of the literature was conducted according to the Preferred Reporting Items for Systematic Reviews and Meta-Analyses guidelines on adult endogenous ACPCs. The review protocol was prospectively registered with PROSPERO (registration number CRD42020184775). The electronic databases of EMBASE and PubMed were searched using the following search terms: (cartilage AND (articular OR hyaline OR knee OR hip OR ankle)) AND (progenitor OR progenitor cell OR multipotent cell OR chondroprogenitor OR multipotent cell OR cartilage-derived OR articular cartilage-derived OR (stem cell OR MSC OR mesenchymal stem cell OR mesenchymal stromal cell AND (cartilage-derived OR cartilage resident))). A final search was performed on 17 February 2021. Two authors (M.R. and J.V.K.) independently screened all selected studies for eligibility, first by title and abstract followed by full-text screening. After duplicate removal, inconsistencies between the researchers were discussed in a consensus meeting.

### Inclusion and exclusion criteria

Inclusion criteria that were used during the title, abstract, and full-text screening for eligible studies included: adult endogenous cartilage stem/progenitor cells; knee, hip, or ankle cartilage; in vitro and/or in vivo and/or in man studies; English language; reviews, case reports, conference papers, studies of which the full texts were not retrievable, studies investigating cell line of chondroprogenitors, cells other than endogenous cartilage-derived progenitors, and lineage-tracing studies were excluded. Extracted data from the selected studies included species, anatomical location of cartilage, isolation procedure, cell characterization, and application of the cells. The quality of a study was considered inferior if methods or results are poorly reported. Study limitations/inconsistencies are discussed at the end of a paragraph in the results.

## Data Availability

Data sharing is not applicable to this article as no datasets were generated or analyzed during the current study.
